# Predictive score of sarcopenia occurrence one year after bariatric surgery in severely obese patients

**DOI:** 10.1371/journal.pone.0197248

**Published:** 2018-05-14

**Authors:** Cosmin Sebastian Voican, Amandine Lebrun, Sophie Maitre, Panagiotis Lainas, Karima Lamouri, Micheline Njike-Nakseu, Martin Gaillard, Hadrien Tranchart, Axel Balian, Ibrahim Dagher, Gabriel Perlemuter, Sylvie Naveau

**Affiliations:** 1 Faculté de Médecine Paris-Sud, Univ Paris-Sud, Université Paris-Saclay, Le Kremlin-Bicêtre, France; 2 INSERM U996, DHU Hepatinov, Labex LERMIT, Clamart, France; 3 Service d’Hépato-Gastroentérologie et Nutrition, Hôpital Antoine-Béclère, Hôpitaux Universitaires Paris-Sud, Assistance Publique-Hôpitaux de Paris, Clamart, France; 4 Service de radiologie, Hôpital Antoine-Béclère, Hôpitaux Universitaires Paris-Sud, Assistance Publique-Hôpitaux de Paris, Clamart, France; 5 Service de Chirurgie Digestive Minimale Invasive, Hôpital Antoine-Béclère, Hôpitaux Universitaires Paris-Sud, Assistance Publique-Hôpitaux de Paris, Clamart, France; McMaster University, CANADA

## Abstract

**Background and aim:**

Sarcopenic obesity is a risk factor of morbidity and mortality. The aim of this study was to generate a predictive score of sarcopenia occurrence one year after bariatric surgery.

**Patients and methods:**

We conducted an observational prospective cohort study on a total of 184 severely obese patients admitted to our institution to undergo sleeve gastrectomy. Skeletal muscle cross-sectional area at the third lumbar vertebrae (SMA, cm^2^) was measured from the routinely performed computed tomography. The skeletal muscle index (SMI) was calculated as follows: SMA/height^2^ (cm^2^/m^2^). Sarcopenia was defined as an SMI < 38.5 cm^2^/m^2^ for women and < 52.4 cm^2^/m^2^ for men. Measurements were performed at surgery and one year later.

**Results:**

Most of the included patients were female (79%), with a mean age of 42±0.9 years and body mass index of 43.2±0.5 kg/m^2^. Fifteen patients (8%) had sarcopenia before surgery and 59 (32%) at the one-year follow-up. Male gender (p<0.0001), SMA before surgery (p<0.0001), and SMI before surgery (p<0.0001) significantly correlated with the occurrence of sarcopenia one year after surgery by multivariate analysis. Two predictive sarcopenia occurrence scores were constructed using SMA and gender (*SS1 score*) or SMI and gender (*SS2 score*). The area under receiver operating characteristic (AUROC) curve of the *SS2 score* was significantly greater than that of the *SS1 score* for the diagnosis of postoperative sarcopenia occurrence (0.95±0.02 *versus* 0.90±0.02; p<0.01). A cut-off value for the *SS2 score* of 0.53 had a sensitivity of 90%, a specificity of 91%, a positive predictive value of 83%, and a negative predictive value of 95%. In the group of patients without baseline sarcopenia, the *SS2 score* had still an excellent AUROC of 0.92±0.02. A cut-off of 0.55 predicted development of sarcopenia one year after sleeve gastrectomy in these patients with a sensitivity of 87%, a specificity of 88%, and negative predictive value of 95%.

**Conclusion:**

The *SS2 score* has excellent predictive value for the occurrence of sarcopenia one year after sleeve gastrectomy. This score can be used to target early intensification of nutritional and dietetic follow-up to the predicted high-risk population.

## Introduction

Sarcopenia is a pathological disorder characterized by generalized loss of skeletal muscle mass and function. It is a risk factor of physical disability, impaired quality of life, and death [1, 2]. Sarcopenia is also associated with diabetes, metabolic syndrome, and cardiovascular disease [3]. Furthermore, sarcopenia is associated with the severity of fibrosis and steatosis, independently of metabolic risk factors, in patients with nonalcoholic fatty liver disease (NAFLD) [4]. Analysis of single cross-sectional computerized tomography scan (CT-scan) images, acquired during routine care, is increasingly being used to quantify whole-body muscle mass in vivo. The skeletal muscle cross-sectional area measured on CT-scan images at the third lumbar vertebrae (SMA, cm^2^) is linearly related to whole-body muscle mass [5, 6]. Therefore, SMA normalized for stature, called skeletal muscle index (SMI, cm^2^/m^2^), is used to predict whole-body muscle mass. Prado *et al*. [7] determined sex-specific SMI cut-offs to define sarcopenia in obese patients with solid tumors of the respiratory and gastrointestinal tracts. Men with an SMI < 52.4 cm^2^/m^2^ and women with an SMI < 38.5 cm^2^/m^2^ are sarcopenic and have a poorer functional status and higher mortality [7]. These cut-points have further been linked to adverse outcomes in various populations including the intensive care unit, cancer and liver disease patients [8–10].

Sarcopenic obesity is defined as the co-presence of sarcopenia and obesity. Its burden is likely to increase worldwide due to the increasing prevalence of obesity [2, 7, 11]. Bariatric surgery is an efficient treatment for severe obesity, resulting in significant weight loss and decreased obesity-associated comorbidity. The repercussions of major weight loss following bariatric surgery on the onset of sarcopenia or modification of pre-existing sarcopenia are poorly documented. Furthermore, early instauration of adequate nutritional support in combination with physical activity is a major anabolic stimulus for muscle protein synthesis and prevention of sarcopenia occurrence [12]. The aim of this study was to define a predictive score of sarcopenia occurrence in a large observational prospective cohort of severely obese patients one year after sleeve gastrectomy.

## Patients and methods

### Study population

We conducted an observational prospective cohort study including consecutive patients admitted to our institution to undergo sleeve gastrectomy were prospectively included between January 2013 and January 2014. Patients were eligible for inclusion if they (1) were severely obese [body mass index (BMI) ≥ 35 kg/m^2^] with comorbid conditions or morbid obesity alone (BMI ≥ 40 kg/m^2^) and were not responding to medical treatment; (2) had no medical or psychological contraindications for bariatric surgery; (3) had no previous bariatric surgery; (4) had perioperative and postoperative (at one year) evaluation (clinical, biological, and computed tomography (CT) scan); and (5) were 18 years of age or older. The CT scans were performed two days and one year after sleeve gastrectomy. All patients underwent a laparoscopic single-port sleeve gastrectomy. Patients were excluded if they did not have a CT scan between 12 and 18 months after surgery. Written informed consent was obtained from all participants. The study did not included minors and was conducted in accordance with the French law concerning medical investigations (Huriet Law) and the Helsinki declaration. The study protocol and the consent procedure were approved by the ethics committee of the Bicêtre Hospital.

### Computed tomography

The skeletal muscle cross-sectional area at the third lumbar vertebrae (SMA, cm^2^) was measured by CT scan according to the method of Prado *et al*. [7]. The SMA was identified and quantified using Hounsfield unit thresholds (-29 to +150) [5, 13]. The third lumbar vertebrae region contains psoas, paraspinal muscles (quadratus lumborum and erectorspinae), and abdominal wall muscles (external and internalobliques, transversus abdominus, and rectus abdominus). Measurements were performed by a single reader by delineating the muscle area. The SMI (cm^2^/m^2^) was calculated as follows: SMA/[height (m) × height (m)]. Sarcopenia was defined as a SMI < 38.5 cm^2^/m^2^ for women and < 52.4 cm^2^/m^2^ for men [7]. The fat-free mass (FFM) was estimated by the following regression equation previously validated by Moutzarkis *et al*. [6]: FFM (kg) = 0.3×SMA(cm^2^) + 6.06. CT scans were systematically performed two days and one year after sleeve gastrectomy for early and late surgical complication screening. The time between the two scans was between 12 and 18 months. The clinical interest of routine postoperative CT scan was evaluated by our team in a previous study [14]. We showed that a combination of clinical surveillance and early imaging allowed prompt management of complicated cases, avoiding further morbidity. The potential risks associated with diagnostic medical radiation should always be considered. Bariatric surgery patients may receive radiation doses from postoperative radiological exams that could potentially increase their lifetime risk of cancer. Although the risk to benefit ratio may be justified, strategies to minimize the radiation dose in this patient population should be applied. In our patients, CT scan was performed on a targeted zone of interest using a limited number of phases, thus considerably decreasing radiation exposure.

### Serum biological markers

The following parameters were assessed prospectively on fresh serum: alanine aminotransferase (ALT), aspartate aminotransferase (AST), gamma glutamyl transpeptidase (GGT), fasting blood glucose, glycosylated hemoglobin (HbA1c), cholesterol, triglyceride, LDL-cholesterol, HDL-cholesterol, ferritin, C reactive protein (CRP), hemoglobin, creatinine, and albumin levels, and prothrombin time.

### Statistical analysis

Qualitative variables were compared using chi-square and Fisher exact tests, as appropriate. Student’s t-test was used for comparisons of normally distributed quantitative variables, and the Mann–Whitney or Kruskal–Wallis test to compare quantitative variables that were not normally distributed. Quantitative variables are expressed as the mean ± SEM. The Bonferroni multiple-comparison procedure was used. Univariate analysis of baseline clinico-biological parameters was carried out for patients developing sarcopenia one year after sleeve gastrectomy *versus* those who did not. We then identified independent relationships between variables with a P value < 0.05 by univariate analysis and the presence of sarcopenia one year after sleeve gastrectomy, through logistic regression analysis. The variables with P < 0.05 by multivariate analysis were retained to generate two logistic regression functions for predicting the occurrence of sarcopenia one year after sleeve gastrectomy, called the *SS1 score* and the *SS2 score*. The best index for predicting the occurrence of sarcopenia was a logistic regression function that combined gender and SMA (*SS1 score*) or gender and SMI (*SS2 score*): XB = α+∑β_i_x_i_ (α = intercept; β_i_ = regression coefficients for x_i_; x_i_ = explanatory variables). Each model estimates B for a specific group, where Logit (Y) = XB. The group probability score was calculated by transforming the logit function using Prob(Y = sarcopenia) = 1/(1+Exp(-XB). The probability Y of sarcopenia is a value between 0 and 1. The overall diagnostic performance of each sarcopenia score (*SS1* and *SS2 scores*) was determined using areas under receiver operating characteristic (AUROC) curves with an empirical nonparametric method, as described by DeLong *et al*. [15]. Comparisons were made as described by Zhou et al. [16]. Sensitivity, specificity, and positive and negative predictive values were calculated. The optimal cut-off values for each sarcopenia score were determined by maximizing the sum of sensitivity and specificity. We used Number Cruncher Statistical Systems software, version 9.0.14 [17].

## Results

### Characteristics of the patients

A total of 229 consecutive severely obese patients referred to our hospital for sleeve gastrectomy were evaluated between January 2013 and January 2014. Forty-five patients were excluded due to an early follow-up evaluation between three and nine months after surgery (25 patients), loss to follow-up (10 patients), an incomplete evaluation one year after surgery (eight patients), and death (two patients). Finally, 184 patients were included in the analysis. The characteristics of the non-included patients were not significantly different. Most of the included patients were female (79%), the mean age was 42 ± 0.9 years, and the mean BMI was 43.2 ± 0.5 kg/m^2^ (Table 1). The interval between the two CT scan evaluations was 384 ± 5 days. Fifteen patients (8%) had sarcopenia at inclusion and 59 (32%) one year after sleeve gastrectomy. Patients having sarcopenia at inclusion were not significantly different from those without sarcopenia in terms of age, BMI, comorbidities or biology parameters. The characteristics of the included patients are reported in Table 1.

**Table 1:** Characteristics of included patients.

Characteristics	Included (n = 184)
**Baseline**	
**Age (years)**	42±0.9
**Male**	38 (20.7)
**BMI (kg/m**^**2**^**)**	43.2±0.5
**Type 2 diabetes**	35 (19)
**Hypertension**	60 (32.6)
**Hyperlipidemia**	52 (28.3)
**Severe obstructive sleep apnea**	92 (50.3)
**SMA (cm^2^)**	46.3±1.7
**SMI (cm**^**2**^**/m**^**2**^**)**	53.2±0.7
**Calculated FFM (kg)**	50.2±0.8
**Sarcopenia**	15 (8.2)
**ALT (IU/L)**	39±2.1
**AST (IU/L)**	30±1.5
**GGT (IU/L)**	48±5.4
**Fasting blood glucose (mmol/L)**	6±0.3
**HbA1c (%)**	6±0.3
**Cholesterol (mmol/L)**	5.21±0.07
**Serum triglycerides (g/L)**	1.40±0.06
**LDL-cholesterol (mmol/L)**	3.26±0.07
**HDL-cholesterol (mmol/L)**	1.27±0.02
**Ferritin (μg/L)**	111±9.5
**C reactive protein (mg/L)**	6.9± 0.5
**Prothrombin Index (%)**	96±0.9
**Hemoglobin (g/100ml)**	14.1±0.5
**Serum albumin (g/L)**	40.8±0.2
**Hepatic steatosis**	156 (84.5)
**Cirrhosis**	1 (0.5)
**One-year follow-up**	
**BMI (kg/m^2^)**	31.7±0.5
**Weight loss (% of initial weight)**	28±0.7
**SMA (cm^2^)**	124.5±2.2
**SMI (cm**^**2**^**/m**^**2**^**)**	45.1±0.6
**Calculated FFM (kg)**	43.4±0.7
**Sarcopenia**	59 (32.1)

**Note**: Results are shown as the mean ± standard error of the mean or n (%).

**Abbreviations**: BMI, body mass index; SMA, skeletal muscle area; SMI, skeletal muscle index; FFM, fat-free mass; AST, aspartate aminotransferase; ALT, alanine aminotransferase: GGT, gamma glutamyl transferase; HbA1c, glycated hemoglobin; LDL, low density lipoprotein; HDL, high density lipoprotein.

### Univariate analysis according to the occurrence of sarcopenia at the one-year follow-up

We compared the baseline clinico-biological parameters of sarcopenic and non-sarcopenic patients one year following surgery (Table 2). Patients with sarcopenia at the one-year follow-up had a lower baseline BMI, SMA, SMI, and calculated FFM. Being male and having sarcopenia at baseline were more frequent in the group of sarcopenic than non-sarcopenic patients one year after sleeve gastrectomy. The other clinico-biological parameters were not significantly different between the two groups (Table 2).

**Table 2:** Univariate analysis of baseline (before surgery) and follow-up (one year after surgery) parameters according to the presence of sarcopenia one year after sleeve gastrectomy.

	Sarcopenia (n = 59)	No sarcopenia (n = 125)	p	
**Baseline**				
**Age (years)**	41.8±1.6	42±1.1	0.75	
**Male (%)**	24 (40.7)	14 (11.2)	<0.0001	
**Type 2 diabetes (%)**	9 (15.3)	26 (20.8)	0.37	
**Hypertension (%)**	16 (27.1)	44 (35.2)	0.28	
**Hyperlipidemia (%)**	14 (23.7)	38 (30.4)	0.35	
**Severe obstructive sleep apnea (%)**	26 (44.1)	66 (53.2)	0.25	
**BMI (kg/m**^**2**^**)**	41.1±0.9	44.1±0,6	<0.0001	
**SMA (cm^2^)**	138±4.3	151.2±3	<0.001	
**SMI (cm**^**2**^**/m**^**2**^**)**	47.5±1.1	55.9±0.7	<0.0001	
**Calculated FFM (kg)**	47.5±1.3	51.4±0.9	<0.01	
**Presence of sarcopenia (%)**	15 (25.4)	0 (0)	<0.0001	
**ALT (IU/L)**	40.2±3.7	38.7±2.5	0.48	
**AST (IU/L)**	29±2.6	31±1.8	0.89	
**GGT (IU/L)**	48.3±9.6	448±6.6	0.17	
**Fasting blood glucose (mmol/L)**	5.7±0.5	6.2±0.3	0.31	
**HbA1c (%)**	5.6±0.5	6.2±0.4	0.06	
**Cholesterol (mmol/L)**	5.24±0.13	5.20±0.09	0.94	
**Serum triglycerides (g/L)**	1.35±0.11	1.44±0.08	0.52	
**LDL-cholesterol (mmol/L)**	3.24±0.12	3.26±0.08	0.82	
**HDL-cholesterol (mmol/L)**	1.33±0.04	1.25±0.03	0.17	
**Ferritin (μg/L)**	114.2±17	110±12	0.74	
**C reactive protein (mg/L)**	15±1.2	15±0.8	0.92	
**Prothrombin Index (%)**	95.5±1.5	96.3±1	0.20	
**Hemoglobin (g/100ml)**	14.1 ± 0.8	14.2±0.6	<0.05	
**Creatinine (μmol/L)**	80.3±2.5	75.2±1.8	0.24	
**Serum albumin (g/L)**	41±0.4	40.7±0.2	0.25	
**One-year follow-up**				
**BMI (kg/m**^**2**^**)**	28.8±0.8	33±0.5	<0.001	
**Weight loss (% of initial weight)**	30.2±1.2	26.9±0.8	<0.05	
**SMA (cm^2^)**	115.1±3.8	129±2.6	<0.0001	
**SMI (cm**^**2**^**/m**^**2**^**)**	39.5±1	47.7±0.7	<0.0001	
**Calculated FFM (kg)**	40.6±1.1	44.8 0.8	<0.0001	
**Mean delay between the two CT scan (days)**	387±5	384±8	0.66	
**Sarcopenia score 1 (SS1)**	0.75±0.03	0.26±0.02	<0.0001	
**Sarcopenia score 2 (SS2)**	0.82±0.03	0.19±0.02	<0.0001	

**Note**: Results are shown as the mean ± standard error of the mean or n (%).

**Abbreviations**: BMI, body mass index; SMA, skeletal muscle area; SMI, skeletal muscle index; FFM, fat-free mass; AST, aspartate aminotransferase; ALT, alanine aminotransferase: GGT, gamma glutamyl transferase; HbA1c, glycated hemoglobin; LDL, low density lipoprotein; HDL, high density lipoprotein.

### Multivariate analysis

A logistic regression model was fitted to test for independent variables (variables with a p < 0.05 by univariate analyses) at baseline related to the presence of sarcopenia one year after sleeve gastrectomy (dependent variable). In the first analysis, variables with a p-value < 0.05 by the univariate comparison were introduced into the model: gender, BMI, and SMA. Male gender independently and positively correlated with sarcopenia (regression coefficient = 8.50; p < 0.0001) and SMA independently and negatively correlated with sarcopenia (regression coefficient = -0.11; p < 0.0001). We then replaced the SMA by the SMI in the second logistic analysis. Independent variables introduced into the model were gender, BMI. and SMI. Male gender still independently and positively correlated with sarcopenia (regression coefficient = 6.84; p < 0.0001) and SMI independently and negatively correlated with sarcopenia (regression coefficient = -0.42; p < 0.0001).

### Construction of *SS1* and *SS2 sarcopenia scores*

A first model combining the SMA at baseline and gender, called the *SS1 score*, was constructed by logistic regression. The equation for the *SS1 score* is: 13.38 + 8.48 × (sex = 1) - 0.11 × SMA at baseline. R2 (the proportion of variation in the dependent variable accounted for by the independent variables) = 0.42. A second model combining the SMI at baseline and gender called the *SS2 score* was also constructed. The equation for the *SS2 score* is: 19.64 + 6.84 x (sex = 1) - 0.42 × SMI at baseline. R2 = 0.55.

### Predictive value of *SS1* and *SS2 scores* for sarcopenia occurrence one year after sleeve gastrectomy

**Fig 1:**
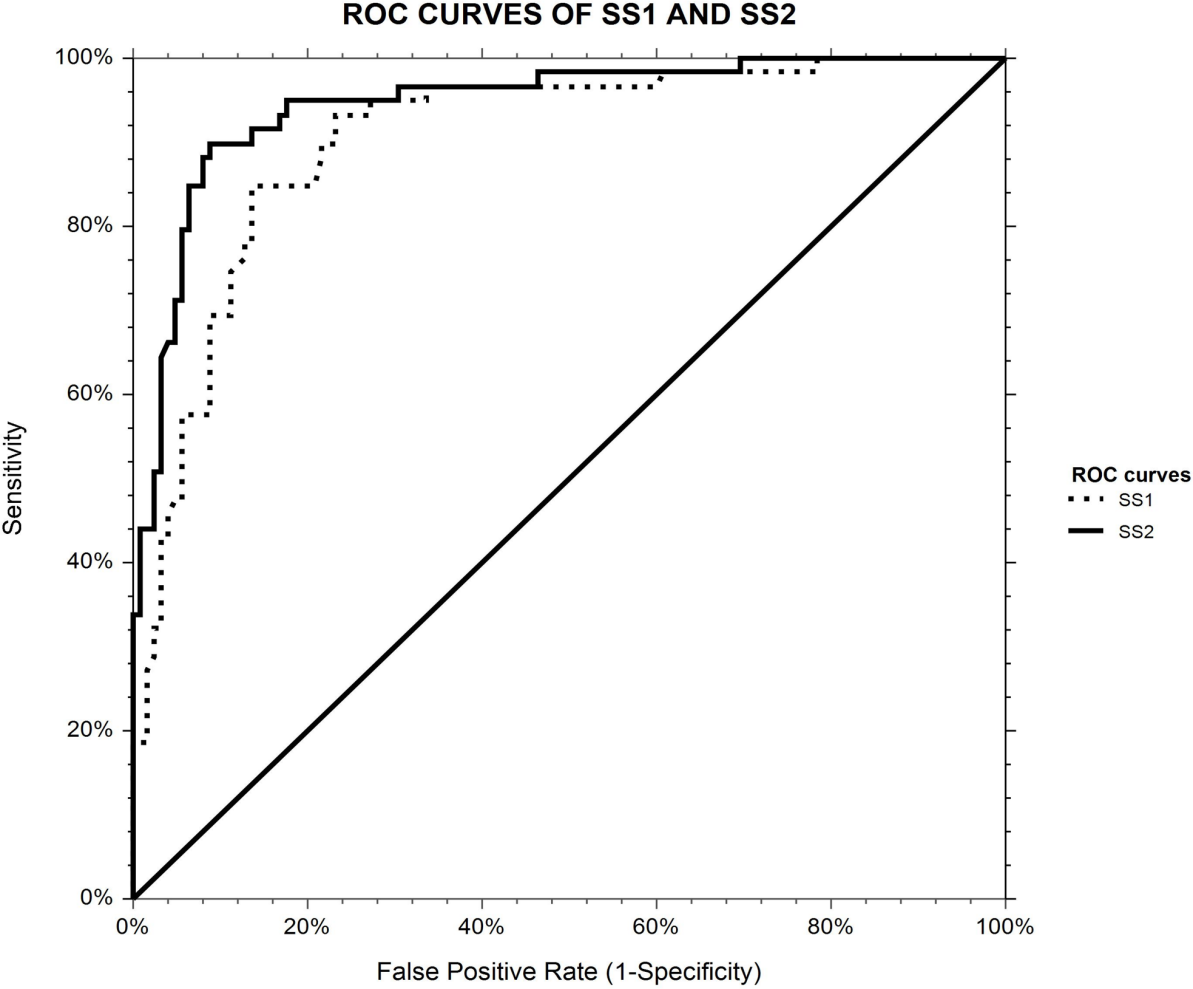
ROC curves of *SS2* and *SS1 scores* for the prediction of sarcopenia one year after sleeve gastrectomy. The diagonal line represents the detection achieved by chance alone (AUROC = 0.50); the ideal AUROC is 1.00. The *SS1 score* takes into account SMA [skeletal muscle cross-sectional area at the third lumbar vertebrae (cm^2^)] and gender. The *SS2 score* takes into account SMI [skeletal muscle index: SMA/height^2^ (cm^2^/m^2^)] and gender.

The AUROC of the *SS2 score* was significantly higher than that of the *SS1 score* for the prediction of sarcopenia one year after sleeve gastrectomy (0.95 ± 0.02 *versus* 0.90 ± 0.02; p < 0.01) (Fig 1). The optimal cut-off for the *SS2 score* was 0.533. This cut-off predicted the presence of sarcopenia one year after sleeve gastrectomy with an excellent sensitivity of 90% and a specificity of 91%, with an excellent negative predictive value (NPV) of 95% and a good positive predictive value (PPV) of 83% (Table 3).

**Table 3:** Predictive values of the *SS2 score* for the presence of sarcopenia one year after sleeve gastrectomy.

*SS2 score* cut-off value	Se	Sp	Prev. PPV	= 0.32 NPV	Prev. PPV	= 0.10 NPV
0.002	1.00	0.14	0.35	1.00	0.11	1.00
0.013	1.00	0.29	0.40	1.00	0.13	1.00
0.052	0.98	0.42	0.45	0.98	0.16	1.00
0.101	0.97	0.56	0.51	0.97	0.20	0.99
0.224	0.95	0.70	0.60	0.97	0.26	0.99
0.403	0.93	0.83	0.72	0.96	0.38	0.99
***0*.*533***	***0*.*90***	***0*.*91***	***0*.*83***	***0*.*95***	***0*.*53***	***0*.*99***
0.606	0.85	0.94	0.86	0.93	0.60	0.98
0.870	0.61	0.97	0.90	0.84	0.68	0.96
0.962	0.36	0.99	0.95	0.77	0.83	0.93

**Note**: The optimal cut-off value for the *SS2 score* (0.533) was determined by maximizing the sum of sensitivity and specificity.

**Abbreviations**: Se, sensitivity; Sp, specificity; Prev, prevalence; PPV, positive predictive value; NPV, negative predictive value.

### Predictive value of *SS2 score* for sarcopenia occurrence one year after sleeve gastrectomy in patients without baseline sarcopenia

Considering that each of the 15 patients with baseline sarcopenia has kept it one year after surgery, we performed a sensitivity analysis for *SS2 score* after removing these patients from the cohort. In univariate analysis, patients developing sarcopenia at one-year follow-up had still a lower baseline BMI, SMA, SMI and calculated FFM. Being male was more frequent in the group of sarcopenic than non-sarcopenic patients one year after sleeve gastrectomy. In multivariate analysis, male gender still independently and positively correlated with sarcopenia development (regression coefficient = 6.67; p < 0.0001) and SMI independently and negatively correlated with sarcopenia development (regression coefficient = -0.41; p < 0.0001). The *SS2 score* was reconstructed in the group of patients without baseline sarcopenia. The equation of this new *SS2 score* is: 19.34 + 6.67 x (sex = 1) - 0.41 × SMI at baseline. The AUROC of *SS2 score* (0.92±0.02) was not significantly different in the group of patients without baseline sarcopenia compared to the entire cohort. The optimal cut-off for the *SS2 score* in patients without baseline sarcopenia was 0.55. This cut-off predicted development of sarcopenia one year after sleeve gastrectomy in patients without baseline sarcopenia with an excellent sensitivity of 87% and specificity of 88%, with an excellent negative predictive value (NPV) of 95% and a good positive predictive value (PPV) of 73%.

## Discussion

In this study of 184 severely obese patients undergoing sleeve gastrectomy, the *SS2 score*, combining gender and skeletal muscle area at the third lumbar vertebrae (transverse CT-scan section), corrected by high (SMI), showed excellent predictive value for the presence of sarcopenia one year after surgery, with an AUROC of 0.95. The prevalence of sarcopenia was 8% at surgery and rose to one third of patients one year after sleeve gastrectomy. Patients with sarcopenia were more often men, had a lower initial BMI, a lower SMI at surgery, and a higher rate of initial sarcopenia than patients without sarcopenia (Table 2). All patients with sarcopenia at baseline presented sarcopenia after one year. The decrease of muscle mass significantly correlated with weight loss as the percentage of initial weight (r = 0.27, p <0.0001). The prevalence of obese sarcopenia is estimated to be 2% for patients aged 60 to 69 years and increases to 10% for patients aged over 80 years [18]. Eight percent of the patients in our cohort were already sarcopenic before bariatric surgery, despite a low mean age of only 42 years. Previous studies showed that age >75 years is significantly associated with sarcopenia in both men and women [19]. In our cohort, patient age was not an independent predictive factor of sarcopenia occurrence one year after surgery. This discrepancy is probably due to the young age of our patients.

We showed here that male gender and baseline SMI were two independent predictive factors of sarcopenia one year after surgery. These two variables accounted for 55% of the risk of having sarcopenia. The score that we propose is thus generated from gender and SMI, calculated from the SMA at L3 by CT scan. The mechanism of gender-associated risk of developing sarcopenia after bariatric surgery is unknown. In our cohort, males did not differed from females in term of baseline age, BMI or comorbidities (HTA, diabetes, obstructive sleep apnea). In rodent models of high-fat diet, males were more prone to the decline of muscle during ageing than females [20]. In a cohort of patients with lung cancer, a significantly higher proportion of men were sarcopenic, according to CT-scan criteria, compared with women [21]. Nevertheless, association between male gender and sarcopenia development has to be confirmed in bariatric population.

Sarcopenia was highly associated with a low initial skeletal muscle mass and the magnitude of weight loss following sleeve gastrectomy. A previous study evaluating body composition after various types of bariatric surgery showed that the loss of FFM after surgery was independent of the initial BMI and the magnitude of weight loss did not correlate with FFM loss [22], in accordance with our data.

In non-obese patients, sarcopenia is a significant risk factor of frailty, weakness, functional disability, and institutionalization [23–26]. Moreover, sarcopenic obesity impairs functional activities and abilities [7, 27, 28]. In a cirrhotic population, the mortality rate reached 36% in the obesity group, 48% in the sarcopenia group, and 67% in the sarcopenic obesity group [28]. Furthermore, sarcopenic obesity increases the rate of adverse outcomes, including postoperative infections in patients undergoing cardiac surgery (OR: 6.6, 95% CI: 1.7–25.2, p = 0.01) [27]. The survival of sarcopenic obesity patients was 10.0 months shorter than those who were not sarcopenic for patients with solid tumors of the respiratory and gastrointestinal tracts (HR: 4.2, 95% CI: 2.4–7.2, p < 0.0001) [7]. Although the impact of sarcopenia on long-term prognosis in patients with bariatric surgery is unknown, patients with a high risk of developing sarcopenia should probably be identified early to intensify the nutritional and dietetic follow up.

We propose a simple and accurate method to generate a sarcopenia risk score, with excellent predictive performance, using the SMA measured by CT scan two days and one year after sleeve gastrectomy for early and late surgical complication screening. A cut-off of 0.533 for the *SS2 score* for the prediction of one-year post-surgery sarcopenia had an excellent NPV of 0.95 and a good PPV of 0.83. The *SS2 score* had similar diagnosis performance even after excluding patients with baseline sarcopenia. Altogether, these data suggest that the *SS2 score* is a reliable tool to select patients at risk of developing sarcopenia after bariatric surgery. As reported in the literature, the measured SMA correlates with the FFM [5, 7, 13]. DXA is the most commonly used imaging technique to measure FFM because of its precision and tolerance by patients, but it is not widely available [25, 29]. Mitsiopoulos *et al*. compared arm and leg skeletal muscle cross sectional area using magnetic resonance imaging (MRI) and CT scan with corresponding cadaver estimates [13]. Both MRI and CT scan estimates of SMA highly correlated with the corresponding cadaver values [13]. Shen *et al*. showed a high correlation between body composition estimated by whole body multi-slice CT or MRI and single abdominal cross-sectional image [5]. The highest correlation between a single slice SMA and total body skeletal muscle volume was specifically observed 5 cm above the L4-L5 vertebral level [5]. Mourtzaki *et al*. showed that regional analysis of fat and fat-free tissue at L3, by either DXA or CT, strongly correlated with whole-body fat and FFM (r = 0.86–0.94; p < 0.001) [6].

Sarcopenia has been defined by Rosenberg *et al*. as the loss of both muscle mass and function [30]. Recently the International Working Group on Sarcopenia recommended MRI and CT scan as “gold standard” imaging techniques to assess sarcopenia [29]. The European Working Group on Sarcopenia in Older People recommends MRI and CT scan for the measurement of muscle mass to define sarcopenia and the use of cut-off values reported in the reference studies [2]. Prado *et al*. validated the sarcopenia diagnosis with the CT measurement of the skeletal muscle cross-sectional area at L3 [7].

Our study has several limitations. First, the rate of loss to follow up was 20% in this cohort. The non-interventional character of the study can explain the high proportion of patients lost to follow-up. Nevertheless, patients were lost to follow up independently of the evolution of their health and were not significantly different from those who had a one-year post-surgery evaluation. Second, despite excellent diagnostic performance for predicting the presence of sarcopenia one year after sleeve gastrectomy, our score needs validation in an independent external prospective cohort. Third, muscle function tests (such as hand grip strength test) were not available for our patients. Studies evaluating both muscles mass on CT-scan images and function are further needed.

In conclusion, the results of our study show that the *SS2 score*, combining gender and SMI calculated from SMA at L3 on early postoperative CT scan, is a reliable tool for predicting the presence of sarcopenia one year after sleeve gastrectomy. The *SS2 score* should be used to select patients at risk of developing sarcopenia after bariatric surgery to intensify nutritional intervention. Further studies are necessary to validate this predictive score in an independent prospective external cohort and evaluate its long-term prognostic value.
